# Information Processing Mechanisms Associated With Intention to Use Public Reporting Systems: Cross-Sectional Study

**DOI:** 10.2196/105637

**Published:** 2026-11-11

**Authors:** Lanmei Du

**Affiliations:** 1 Department of Management and Entrepreneurship, Turku School of Economics, University of Turku, Rehtorinpellonkatu 3, Turku, 20500, Finland, +358 417296016

**Keywords:** public reporting systems, health care information, information processing, elaboration likelihood model, negative emotional response

## Abstract

**Background:**

Health care public reporting (PR) systems are intended to reduce information asymmetry and support informed health care decision-making. However, their adoption remains limited, partly because health care information is complex, difficult to interpret, and may evoke cognitive and emotional responses. Existing research has focused largely on system design and development, with limited attention to how users’ central and peripheral-route information-processing mechanisms shape PR system adoption.

**Objective:**

This study aims to identify the factors influencing users’ intentions to use health care PR systems by integrating sociotechnical and information-processing perspectives. It specifically examines how perceived information accessibility (PIA), visual information presentation (VIP), health information comprehension (HIC), information credibility (IC), heuristic trust (HT), and negative emotional response (NER) contribute to system adoption.

**Methods:**

We analyzed data from an online questionnaire survey of 315 users in China conducted between October 2025 and May 2026. Partial least squares structural equation modeling (PLS-SEM) with 5000 bootstrap resamples was used to examine the hypothesized relationships among PIA, VIP, HIC, IC, HT, NER, and intention to use (IU).

**Results:**

PIA positively influenced VIP (β=0.455, 95% CI 0.361-0.545), which was positively associated with HIC (β=0.508, 95% CI 0.411-0.596). HIC was positively associated with IC (β=0.494, 95% CI 0.405-0.581), which was the strongest direct predictor of IU (β=0.366, 95% CI 0.269-0.467). HT (β=0.286, 95% CI 0.195-0.380) and NER (β=0.205, 95% CI 0.094-0.317) were also positively associated with IU. A significant sequential mediation pathway was identified from PIA through VIP, HIC, and IC to IU (β=0.042, 95% CI 0.023‐0.069).

**Conclusions:**

Adoption of the health care PR systems is shaped by complementary cognitive, heuristic, and affective information-processing mechanisms. PIA facilitates comprehension and credibility, while HT provides an additional pathway for system adoption. Notably, NERs were positively associated with the IU, contrary to the hypothesized negative relationship. This finding suggests that negative emotions may motivate engagement with health care information rather than simply inhibit adoption. These findings highlight the importance of designing PR systems that are accessible, comprehensible, credible, and responsive to users’ cognitive and emotional needs.

## Introduction

### Background

Health information has long been one of the most frequently searched topics on the internet. Prior research suggests that this trend reflects users’ increasingly active role as “information connoisseurs” who search, compare, and evaluate online health content [1]. Information and communication technologies, including the internet, online communities, and social media, have become key channels for disseminating and exchanging health information and facilitating user interactions [2].

Unlike general information, health information carries unique implications for decision-making, as it is often complex, uncertain, and consequential for individuals [3]. Prior research in information systems further suggests that individuals’ postadoption behaviors are shaped by both internal cognitive processes and external contextual triggers [4,5]. From a system-level perspective, health care policy frameworks such as health technology assessment emphasize that health care innovations should improve patient outcomes without creating additional burdens on users [6].

Because reported health information is derived from underlying health care data [7,8], concerns about information quality inevitably raise questions about the accuracy and reliability of the underlying data [9]. However, the potential for “metric-driven harm” and other unintended consequences associated with public reporting (PR) systems remain unexplored [10].

For instance, reporting bias, data distortion [11], and inaccurate outcome data may undermine the effectiveness of PR systems [12], while inaccurate reporting may also increase patient anxiety [13,14]. A deeper understanding of these issues is crucial to ensuring that PR systems improve health care decision-making without creating unintended harm.

Prior research on PR has primarily focused on system development [15], web development [16], website information display [17], and information presentation formats [18,19]. Studies have also examined data accuracy and data consistency [20,21]. However, the adoption of health care PR systems remains low in practice [22]. In particular, in the current digital environment, the growing use of dashboards and data visualization tools has further heightened concerns about how users interpret and evaluate reported health care data [23,24].

PR influences health care quality through a selection pathway, whereby patients select higher-performing providers based on publicly available information [25]. This process depends not only on the availability of information but also on how users process and evaluate it. Accordingly, this study addresses the following research questions (RQs):

1. RQ1: How does information shape users’ perceptions of and responses to information presented in health care PR systems?
2. RQ2: How do central and peripheral route-processing mechanisms shape users’ intention to use health care PR systems?

Guided by these questions, we examine how perceived information accessibility (PIA), health information comprehension (HIC), information credibility (IC), negative emotional response (NER), visual information presentation (VIP), and heuristic trust (HT) contribute to users’ intentions to use health care PR systems.

### Related Work and Model Development

#### Sociotechnical Systems Approach

PR is intended to reduce information asymmetries and enable patients, families, and payers to make more informed choices among health care providers [26]. From a sociotechnical perspective, system effectiveness depends not only on technical design but also on organizational behavior and user-level interpretation processes [27,28]. However, empirical evidence suggests that the impact of the PR system on health care decision-making remains limited [22], indicating that making information publicly available does not necessarily ensure that users can effectively use it.

This limitation highlights the importance of examining information processing at the individual level. Users must understand technical terms and complex concepts, compare alternatives across multiple attributes, and weigh trade-offs according to their preferences when interpreting health care information [29].

However, existing PR research has focused predominantly on system-level attributes [10,12], with limited attention to how users’ abilities, prior experience, and skills shape their processing and use of PR information. This leaves an important gap in explaining differences in users’ cognitive evaluations, attitudes, and behavioral intentions toward PR systems.

Prior research indicates that individual characteristics, including experience, abilities, and skills, influence how people process information [30]. Moreover, greater information acquisition is associated with stronger problem-solving tendencies [31]. These findings suggest that information processing may be an important mechanism through which users engage with information-rich PR systems. Accordingly, this study examines information processing at the individual level to better understand how users interpret and evaluate PR information and how these processes contribute to their intention to use PR systems.

#### Information Processing

Studies have examined factors influencing users’ perceptions and attitudes toward PR systems [22]. However, limited attention has been paid to how users process the information presented by these systems and how cognitive and affective responses jointly shape their intention to use them. Understanding information processing is particularly important in PR contexts, where users may encounter large amounts of complex or potentially conflicting information that can influence both their cognitive evaluations and emotional responses.

Prior research has shown that inconsistencies in reported data can undermine users’ trust in PR systems [32,33], thereby affecting their willingness to use and engage with PR information. Trust has been widely studied across psychology, management, economics, and information systems [34,35]. Beyond trust, however, users’ responses to PR systems may also depend on how they process information and cope with the emotional responses elicited during this process.

To further explain how users process information, this study draws on the elaboration likelihood model (ELM). The ELM proposes that individuals may process information through 2 routes: the central route, which involves careful and effortful evaluation of message content and supporting evidence, and the peripheral route, which relies more on simple cues and heuristics [36,37]. The extent to which individuals engage in more elaborate information processing depends, among other factors, on their motivation and ability to process it. When information is perceived as personally relevant, important, or potentially threatening, individuals may have greater motivation to scrutinize and evaluate it. In contrast, when individuals have limited motivation, knowledge, or processing ability, they may rely more heavily on peripheral cues. These 2 processing routes provide a useful framework for understanding how users evaluate and respond to information presented through PR systems.

In addition to information processing, psychological theories of emotion and control provide further perspectives for understanding users’ responses to information systems. Emotion regulation theory conceptualizes emotion regulation as the set of processes through which individuals influence, maintain, or modify their emotional states. Individuals may engage in various behaviors to manage unpleasant emotional experiences, including seeking information that helps them understand or cope with uncertain situations [38,39]. From this perspective, information systems that provide explanations, guidance, or decision support may serve not only as sources of information but also as resources for managing negative emotional experiences.

The perspective of perceived control further suggests that uncertainty about a situation, its potential consequences, or appropriate courses of action may undermine individuals’ sense of control and generate anxiety [40]. Information seeking can serve as a means of reducing uncertainty and restoring predictability and control. By obtaining additional information, users may develop a clearer understanding of their situation and identify possible actions. This perspective therefore provides a basis for understanding why NERs may coexist with active information-seeking behavior rather than necessarily leading to avoidance.

Taken together, these theoretical perspectives provide a multidimensional framework for understanding users’ responses to PR systems. The ELM explains how users may process information through different routes depending on their motivation and ability, while emotion regulation and perceived control perspectives help explain why users may actively seek information when experiencing NERs or uncertainty. Integrating these perspectives therefore provides a theoretical basis for examining how cognitive and affective responses jointly shape users’ intention to use PR systems.

### Hypotheses Development

PIA refers to users’ subjective perception that information is easy to find, understand, obtain, and use and is therefore a key dimension in evaluating information systems and PR [41]. For patients, existing research on PR has shown that many patients are unaware of the availability of public reporting information [42]. These findings suggest that merely making information available may not be sufficient to ensure that it is accessible to users. The first step in system design is to identify and understand user needs. When the perceived need for a PR system is limited, it may influence the design and development of the system, particularly the way information is presented to users through the web interface.

VIP refers to the quality and characteristics of information as shaped by its format, structure, and presentation, which influence how effectively it can be interpreted and used by recipients [43]. VIP techniques, such as color coding and charts, can reduce the difficulty of information processing and thereby facilitate users’ understanding of PR information [44,45].

HIC, defined as the ability to correctly understand, interpret, and use health-related information [46], plays an important role in shaping users’ perceptions of IC, as users must first understand information before they can evaluate its credibility [42,47,48].

Many hospitals and health care professionals remain skeptical about PR and pay-for-performance programs because of concerns that these programs may mislead patients [49]. Experts have expressed concerns that publicly disclosed outcome data may be misinterpreted or misused, increasing the risks of reporting bias and data distortion [11]. More importantly, concerns about IC may directly affect users’ willingness to use PR systems.

In health care PR, anxiety can be understood as an unintended psychological and behavioral response to publicly disclosed quality, safety, survival, or performance information. Inaccurate reporting of information can lead to patient anxiety, depression, and feelings of hopelessness [13]. Studies show that patients sometimes avoid consulting outcome data when the information is alarming, such as mortality statistics or adverse event rates [50,51].

The peripheral route is for individuals with limited shared history and minimal information about one another (ie, weak ties), who tend to form trust through peripheral cues such as social categories, third-party information, rules, roles, and reputation [52,53]. As patients increasingly seek to actively engage with health care, systems provide a means for information retrieval [54]. Information characteristics may enhance users’ perception of the accuracy and professionalism of publicly reported information [55].

Based on the theoretical arguments and empirical evidence discussed above, the following hypotheses are proposed:

- H1: PIA positively influences VIP.
- H2: VIP influences HIC.
- H3: HIC positively influences IC.
- H4: IC positively influences users’ intention to use PR systems.
- H5: NER negatively influences users’ intention to use PR systems.
- H6: HT positively influences users’ intention to use PR systems.

## Methods

### Study Design and Participants

Health care PR systems provide information about the quality and performance of health care providers and services to support users’ evaluation and decision-making. In this study, we focus on publicly available information used to evaluate health care providers and services on the medical platform (eg, Haodf).

A total of 315 valid responses were collected from participants in China via an online questionnaire-based recruitment process between October 2025 and May 2026. Given the limited research on PR systems in the context of digital health, this study did not impose strict criteria regarding participants’ age or gender.

Participants were informed that all data would be used solely for academic research purposes and would be handled anonymously and confidentially. They were also instructed to answer all questions based on their actual experiences and perceptions.

### Ethical Considerations

The study involved participants who voluntarily completed an anonymous questionnaire addressing the core constructs examined in this research. Prior to participation, respondents received information about the purpose and nature of the study and were informed that their participation was voluntary. Completion of the questionnaire was considered informed consent to participate in the study.

In accordance with Finnish ethical review practices and the Finnish National Board on Research Integrity (Tutkimuseettinen neuvottelukunta [TENK]) guidelines (2019), the researcher assessed the need for a prior ethical review based on the criteria specified in the guidelines. Based on this assessment, the present study did not involve the types of research procedures or circumstances for which a prior ethical review is required under the TENK guidelines [43]. In particular, the study involved no physical or psychological intervention, no manipulation of participants, and no collection of directly identifiable personal information. The study was conducted in accordance with relevant principles of research ethics, including voluntary participation, informed consent, anonymity, confidentiality, and responsible management of research data.

### Data Validity

The questionnaire introduced the context of public health care service platforms and was designed to examine how users process and interpret information provided through PR systems. In this study, health care service providers included hospitals, clinics, and other health care organizations.

Participants were asked whether they had previously used medical website ratings, medical website reviews, or health care service comparison tools. Because China’s health care system is fragmented across multiple relatively independent systems within the broader health care ecosystem, participants who answered “Yes” to any one of these 3 questions were considered eligible and included in the analysis.

### Variables and Measures

Data were collected using a structured survey instrument consisting of 48 items measured on a 7-point Likert scale (1=incompatible, 7=compatible). The questionnaire was designed to assess participants’ perceptions of information presentation, trust dimensions, cognitive processing, and behavioral intentions in relation to PR systems. Detailed variable definitions, questionnaire items, and operationalization procedures are provided in Multimedia Appendix 1.

### Dependent Variable

Regarding the intention to use (IU), the use of intention was measured using a 7-point Likert scale (1=incompatible, 7=compatible). Attitude-behavior relationships were grounded in the works of Davis [56] and Fishbein and Ajzen [57].

### Independent Variables

The measurement of PIA was adapted from the information quality framework proposed by Wang and Strong [58], which conceptualizes accessibility as an important dimension of information quality.

NERs affect behavior [56]. In the context of PR, previous research has shown that metric-driven harm may induce anxiety and fear, which can, in turn, affect users’ actual engagement with and use of PR systems [50,59]. To examine the behavioral effects of NERs in the present study, we drew on the framework linking emotions to behavior [56] and incorporated the peripheral-route perspective of the ELM.

Affective trust in health care refers to the emotional bond that develops between patients and health care providers, based on feelings of care, concern, attention, and emotional engagement that patients experience during interactions, especially in diagnostic and therapeutic processes [60]. It is distinct from cognitive trust, which is based on the patient’s rational assessment of the provider’s competence, reliability, and ethics [61]. In this study, affective trust was mapped onto the construct of HT. HT was assessed using a 7-point Likert-scale item, with the original scale subsequently recoded into 5 categories for analysis.

### Control Variables

A total of 315 valid responses were collected from participants in China through an online, questionnaire-based recruitment process between October 2025 and May 2026. Demographic variables were collected, including gender, age, education level, chronic disease status, prior experience with online health care platforms, and previous use of the medical platform for consultation.

Although some previous studies in the PR literature have examined differences based on users’ gender and age, this study focuses on system development and seeks to identify the needs of actual users of PR systems. Therefore, no further user segmentation was performed based on demographic characteristics. The questionnaire began with a screening question asking whether participants had previously used a health care platform, with responses recorded as binary yes/no (Y=yes, N=no).

### Data Analysis

#### Statistical Analysis and Structural Equation Modeling Specification

Structural equation modeling (SEM), a widely used methodological tool in the social sciences, is used to examine complex relationships between latent constructs and observed variables [62-64]. In this study, SEM was adopted to investigate the factors influencing sustainable system use.

#### Measurement Model Evaluation

The analysis followed a standard procedure. First, the data were screened, with missing values and outliers identified and excluded as appropriate. Second, the measurement model was assessed using Cronbach α and composite reliability (CR), with values above 0.70 indicating acceptable internal consistency. Convergent validity was evaluated using the average variance extracted (AVE), with values exceeding 0.50 considered acceptable.

Discriminant validity was assessed using the Fornell-Larcker criterion and the heterotrait-monotrait (HTMT) ratio. Third, the structural model was estimated using bootstrapping with 5000 resamples to assess path coefficients, *t* values, and *P* values. Finally, predictive accuracy was assessed using the *R*² values of the endogenous constructs.

#### Model Fit Assessment

The explanatory and predictive performance of the partial least squares structural equation modeling (PLS-SEM) structural model was assessed using the coefficient of determination (*R*²), the Q²_predict statistic, and prediction errors based on the PLS-SEM model (partial least squares root mean square error [PLS-RMSE]) and a linear model benchmark (linear model root mean square error [LM-RMSE]). These complementary measures were used to assess the structural model's explanatory and predictive performance (Multimedia Appendix 2).

### Structural Models

#### Overview

By integrating the measurement model and the structural model, SEM enables the validation of measurement instruments while simultaneously examining the hypothesized relationships among constructs.

#### Hypothesized Structural Model

A theory-driven structural model was specified to test the hypothesized paths (H1-H6). PIA, HT, and NER were specified as exogenous theoretical predictors, whereas VIP, HIC, and IC were modeled as endogenous mediators, with IU specified as the endogenous outcome. All model specifications were theory-driven and transparently reported in the final path diagram and supplementary materials.

### Robustness Checks and Supplementary Analyses

As a supplementary analysis, a serial mediation model was estimated to examine whether the association between PIA and IU was sequentially mediated by VIP, HIC, and IC (PIA → VIP → HIC → IC → IU). Indirect effects were assessed using 5000 bootstrap resamples and 95% CIs.

## Results

### Sample Characteristics

Table 1 presents the demographic characteristics of the participants. The age distribution was relatively diverse, with participants aged 25 to 34 years constituting the largest age group, accounting for 77 out of 315 (24.44%) respondents, followed by those aged 18 to 24 years (n=73, 23.17%) and 35 to 44 years (n=69, 21.90%). The gender distribution was relatively balanced, with 168 (53.33%) male participants and 147 (46.67%) female participants.

**Table 1.** Demographics of respondents (N=315).

Demographic	Values, n (%)
Sex	
Male	168 (53.33)
Female	147 (46.67)
Age (y)	
<18	10 (3.17)
18‐24	73 (23.17)
25‐34	77 (24.44)
35‐44	69 (21.9)
45‐54	38 (12.06)
55‐64	39 (12.38)
>60	9 (2.86)
Educational level	
Middle school and below	33 (10.48)
High school	47 (14.92)
Postsecondary	77 (24.44)
College	96 (30.48)
Master	39 (12.38)
Doctoral	23 (7.30)
Chronic disease	
Yes	112 (35.56)
No	203 (64.44)

In terms of educational attainment, 33 (10.48%) participants had a middle school education or below, 47 (14.92%) had completed high school, 77 (24.44%) had postsecondary education, 96 (30.48%) held a college degree, 39 (12.38%) held a master’s degree, and 23 (7.30%) held a doctoral degree. Regarding chronic disease status, 112 (35.56%) participants reported having a chronic disease, whereas 203 (64.44%) participants reported no chronic disease. Since all participants had prior experience using online health care platforms and accessing hospital rating information, the sample provides a relevant representation of users with direct experience with PR systems.

### Data Analysis

Data analysis was conducted in the R (R Foundation for Statistical Computing) statistical environment using the seminar package. Thus, we examined the correlation matrix among constructs [65]. Both the descriptive analyses and the PLS-SEM analysis were conducted using the same analytical sample of 315 respondents. The full collinearity variance inflation factor (VIF) values ranged from 1.456 to 2.074 (Multimedia Appendix 2), remaining well below the commonly recommended threshold of 3.3, thereby indicating that multicollinearity did not pose a serious concern in the model.

### Indicator Reliability and Internal Consistency

The reliability and convergent validity results further support the adequacy of the measurement model [66]. As shown in Table 2, internal consistency was assessed using Cronbach α and CR, while convergent validity was assessed using the AVE and outer loadings.

**Table 2.** Measurement model reliability and convergent validity.

Construct and Items	Loadings	Cronbach α	CR^a^	AVE^b^
NER^c^		0.955	0.963	0.791
NER1	0.979			
NER2	0.850			
NER3	0.902			
NER4	0.871			
NER5	0.858			
NER6	0.890			
NER7	0.866			
HIC^d^		0.940	0.957	0.848
HIC1	0.974			
HIC2	0.909			
HIC3	0.915			
HIC4	0.883			
HT^e^		0.936	0.952	0.797
HT1	0.968			
HT2	0.877			
HT3	0.875			
HT4	0.874			
HT5	0.866			
IC^f^		0.953	0.964	0.842
IC1	0.975			
IC2	0.899			
IC3	0.887			
IC4	0.907			
IC5	0.916			
IU^g^		0.953	0.964	0.844
IU1	0.979			
IU2	0.893			
IU3	0.882			
IU4	0.920			
IU5	0.916			
PIA^h^		0.935	0.949	0.756
PIA1	0.875			
PIA2	0.846			
PIA3	0.871			
PIA4	0.861			
PIA5	0.889			
PIA6	0.874			
VIP^i^		0.944	0.957	0.819
VIP1	0.974			
VIP2	0.897			
VIP3	0.879			
VIP4	0.885			
VIP5	0.885			

a CR: composite reliability.

b AVE: average variance extracted.

c NER: negative emotional response.

d HIC: health information comprehension.

e HT: heuristic trust.

f IC: information credibility.

g IU: intention to use.

h PIA: perceived information accessibility.

i VIP: visual information presentation.

The outer loadings ranged from 0.846 to 0.979, with all indicators exceeding the commonly recommended threshold of 0.70, indicating satisfactory indicator reliability. Cronbach α values ranged from 0.935 to 0.955, while CR values ranged from 0.949 to 0.964, all exceeding the recommended threshold of 0.70 and demonstrating satisfactory internal consistency.

Furthermore, the AVE values ranged from 0.756 to 0.848, exceeding the recommended threshold of 0.50 and thereby confirming adequate convergent validity. Overall, these results indicate that the measurement model demonstrates satisfactory reliability and convergent validity.

Measurement model validity generally consists of 2 dimensions: reliability and validity. Reliability refers to the consistency and stability of the measurement instrument, while validity evaluates whether the constructs accurately capture the concepts they are intended to measure. In this study, reliability was assessed using Cronbach α and CR. The results indicate strong internal consistency across all constructs. Cronbach α values range from 0.935 to 0.955, exceeding the recommended threshold of 0.70. Similarly, CR values range from 0.949 to 0.964, also well above the acceptable level of 0.70, indicating high construct reliability.

Convergent validity was evaluated using the AVE, with a recommended cutoff value of 0.50. The results indicate that all constructs achieve satisfactory convergent validity, with AVE values ranging from 0.756 to 0.848, exceeding the recommended threshold. The item loadings range from 0.846 to 0.979, with the majority of loadings exceeding 0.70. Although several items have loadings below 0.90, the overall AVE values for all constructs remain above the recommended threshold of 0.50, providing evidence of satisfactory convergent validity and adequate item representation of their respective constructs.

### Convergent and Discriminant Validity

Discriminant validity was assessed using the HTMT [67,68]. As shown in Multimedia Appendix 2, all HTMT values were below the conservative threshold of 0.85, indicating adequate discriminant validity among the constructs. Specifically, the highest HTMT value was observed between IC and IU (0.628), followed by HT and VIP (0.609) and HT and IU (0.590). The remaining HTMT values ranged from 0.395 to 0.578, all of which were below the recommended threshold of 0.85. These results confirm that the constructs are empirically distinct and that discriminant validity is established in the measurement model.

### Structural Model Evaluation (H1-H6)

As illustrated in Multimedia Appendix 2 the findings indicate that PIA has a significant positive effect on HIC through VIP (β=0.231). Finally, mediation effects were evaluated using a bootstrapping procedure with 5000 resamples and 95% CIs. As shown in Figure 1, IU is shaped through sequential cognitive and credibility-related pathways, while HT and NER provide additional direct pathways to IU.

**Figure 1.**
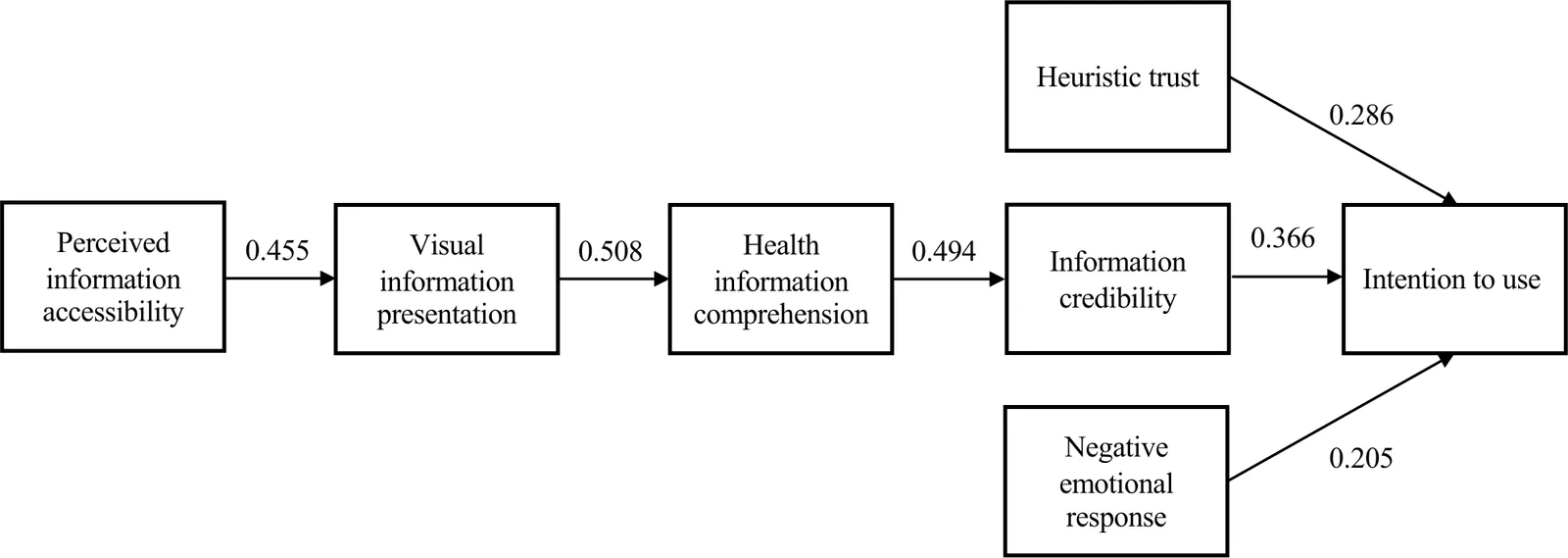
The structural model.

### Hypothesis Validation (H1-H6)

As shown in Table 3, among the antecedents of IU, IC emerged as the strongest direct predictor (β=0.366), followed by HT (β=0.286) and NER (β=0.205). This finding suggests that cognitive understanding is particularly important for IU, while HT and emotional responses also contribute significantly. In addition, HIC functions as an important upstream factor influencing both IC and NER, highlighting its central role in the proposed nomological network.

**Table 3.** Direct path.

Hypotheses	Path	Path coefficient (β, 95% CI)	Mean (SD)	Bootstrap t statistic	*P* value	Effect size (f²)	Result
H1	PIA^a^->VIP^b^	0.455 (0.361-0.545)	0.457 (0.047)	9.677	<.001	0.260	Supported
H2	VIP->HIC^c^	0.508 (0.411-0.596)	0.509 (0.047)	10.732	<.001	0.348	Supported
H3	HIC->IC^d^	0.494 (0.405-0.581)	0.496 (0.045)	10.978	<.001	0.323	Supported
H4	IC->IU^e^	0.366 (0.269-0.467)	0.367 (0.051)	7.219	<.001	0.182	Supported
H5	NER^f^->IU	0.205 (0.094-0.317)	0.205 (0.057)	3.595	<.001	0.056	Not supported; positive effect
H6	HT^g^->IU	0.286 (0.195-0.380)	0.286 (0.047)	6.113	<.001	0.109	Supported

a PIA: perceived information accessibility.

b VIP: visual information presentation.

c HIC: health information comprehension.

d IC: information credibility.

e IU: intention to use.

f NER: negative emotional response.

g HT: heuristic trust.

As shown in Table 3, PIA significantly predicted VIP (β=0.455; *P*<.001), VIP predicted HIC (β=0.508; *P*<.001), and HIC predicted IC (β=0.494; *P*<.001). IC (β=0.366; *P*<.001), HT (β=0.286; *P*<.001), and NER (β=0.205; *P*<.001) positively predicted IU.

The R-squared (*R*²) values of the endogenous constructs were examined to assess the explanatory power of the proposed model. According to Hair et al [65], *R*² values of 0.75, 0.50, and 0.25 can be interpreted as substantial, moderate, and weak explanatory power, respectively. The results indicate that the model explains 25.8% of the variance in HIC (*R*²=0.258), 24.4% of the variance in IC (*R*²=0.244), 20.7% of the variance in VIP (*R*²=0.207), and 48.6% of the variance in IU (*R*²=0.486; Multimedia Appendix 2).

## Discussion

### Interpretation of Principal Findings

This study is grounded in an information-processing perspective [69] and examines how users process information presented through PR systems and subsequently form cognitive, affective, and behavioral responses. The proposed framework distinguishes between system-mediated information processing and human cognitive information processing.

At the system level, PIA reflects the extent to which information can be readily accessed and processed through the PR system. At the user level, information comprehension reflects the subsequent cognitive processing of the presented information. This conceptualization assumes that the way information is presented and made accessible through a digital system can shape how users cognitively process and interpret that information.

Beyond systematic cognitive processing, the PR context may also activate heuristic and affective response mechanisms. Drawing on dual-process perspectives, including the ELM, this study considers cognitive judgments as a central route and NERs as a peripheral route through which users may respond to PR information without relying exclusively on extensive systematic evaluation.

First, the findings support the role of systematic information processing. VIP was positively associated with users’ comprehension of health information, suggesting that information that is easier to access and navigate may facilitate more deliberate processing. This finding is consistent with prior research showing that users’ cognition and awareness of medical information can influence subsequent behavioral responses [42].

In the context of PR systems, greater information accessibility may facilitate better VIP, which in turn supports users’ comprehension of the information. This sequence may subsequently contribute to credibility evaluations and IU. The findings further support the role of the peripheral route described by the ELM; visual and contextual cues may enable users to form judgments about IC without requiring extensive evaluation of the underlying health care indicators.

Elements such as star ratings, colors, charts, and interface layouts can create impressions of professionalism, clarity, and reliability, thereby facilitating the formation of HT [70,71]. This suggests that users do not necessarily need to develop a profound understanding of health care indicators before forming favorable judgments about a PR system. Instead, salient visual cues may serve as cognitive shortcuts that shape perceptions of credibility and, ultimately, intention to use the system.

The finding that NERs are positively associated with IU differs from the commonly observed association between negative emotions and avoidance or disengagement. This finding can be discussed in relation to the theoretical perspectives introduced above. First, the finding is consistent with emotion regulation theory, which emphasizes individuals’ efforts to manage unpleasant emotional experiences through various behaviors, including information seeking [38,39]. Second, it is consistent with the perspective of perceived control, which links uncertainty and anxiety with information seeking as a means of obtaining greater understanding and predictability [40]. Third, the finding can be considered from the ELM perspective, as the model emphasizes the role of motivation and ability in processing personally relevant information [72,73].

Taken together, these perspectives provide complementary theoretical contexts for understanding the positive relationship between NERs and IU. The findings therefore indicate that NERs are not necessarily associated solely with avoidance or disengagement and should also be considered in relation to users’ information-seeking and information-processing responses within PR systems.

### Theoretical Contribution

This study, situated within the context of PR systems, examines how health care information and users’ NERs are associated with system adoption. First, this study integrates the ELM to capture both heuristic and systematic processing of health care information.

Second, this study adopts a dual-process perspective to explain how users process and respond to information presented through PR systems. This perspective distinguishes between systematic processing, in which users engage in more deliberate evaluation of information, and heuristic processing, in which users rely on salient cues and simplified judgments. In the context of PR systems, these processes may operate through different pathways: users may systematically process the accessibility, presentation, comprehension, and credibility of health care information while also relying on visual and contextual cues to form HT. In addition, PR information may elicit affective responses that further influence users’ evaluations and behavioral intentions. By integrating these cognitive, heuristic, and affective mechanisms, this study provides a unified framework for understanding users’ intentions to use PR systems.

Measuring health care quality is difficult, yet it is essential, as improvements in health care quality depend on effective measurement practices [74]. Within collaborative health care governance, prior studies on PR systems have primarily focused on process indicators related to promoting data sharing among diverse stakeholder groups, including physicians, family members, health care organizations, and nurses [17]. Incorporating IC helps explain how users move from processing presented information to forming intentions to use PR systems [75].

Previous research suggested that increasing data transparency and structured presentation can enhance interpretability [71]. In the present study, these information-processing characteristics are further linked to users’ evaluations of IC within PR systems.

In addition, HIC and IC serve as important mediating variables in the proposed model. From a dual-process perspective, these findings suggest that heuristic cues and interpersonal trust may complement users’ more systematic processing of health care information and contribute to system adoption [76]. These findings are consistent with previous research showing that physicians remain one of the most important sources of trust for patients [18,55,77].

### Practical Implications

Previous research has suggested that a unified data-processing framework can help address these challenges by providing an effective means of measuring and monitoring health disparities [78,79]. These findings further underscore the value of integrated data-processing frameworks in supporting IC and collaborative health care governance.

In the digital era, health care can hardly be discussed independent of media and technological mediation [80], and the use of media inevitably exposes users to health care data. Although traditional media environments (eg, books and academic articles) have long relied on peer review to ensure content quality, the emergence of the internet has enabled the unrestricted publication of information by virtually anyone [81].

Previous studies have highlighted the importance of adequately providing personalized care, such as emotional support and pain management [82]. In the context of physical pain and illness, patients may process information differently from healthy individuals [83]. For example, under pain-related stimulation, the prefrontal cortex may not fully perform its normal cognitive processing functions, increasing the importance of systems as mediating tools that help patients participate in the treatment process [84].

### Limitations

This study has several limitations. First, the study relies on self-reported survey data collected through online recruitment, which may introduce common method bias. In addition, due to privacy and data protection considerations, personally identifiable information, such as names, telephone numbers, and email addresses, was neither collected for statistical analysis nor included in the analysis. Future research could use multiple data sources or adopt longitudinal and time-lagged study designs, with data collected at different time points, to further reduce this potential bias.

Second, although the proposed model incorporates several cognitive, affective, and heuristic factors, other potentially relevant variables were not measured. Factors such as health literacy, prior health care experience, digital literacy, and individual health status may influence users’ perceptions and intentions to use PR systems. Research has explored which quality indicators patients prioritize when choosing hospitals and doctors [85]. Future research should place greater emphasis on patient populations, as patients are often considered a vulnerable group [86].

Third, the NER construct integrates several negative emotional states, including frustration, anxiety, uncertainty, and stress, into a single construct. However, the present study did not directly measure anxiety-driven search behavior. In addition, other emotional responses, such as joy, satisfaction, and hope, may also influence users’ intentions to use the system. Future research should distinguish among different emotional states and examine whether they have differential effects on system use.

Fourth, the wording of the survey items may have introduced wording or framing effects. The constructs were originally developed from previous literature in English (Multimedia Appendix 1) and subsequently translated into Chinese. A pretest involving 200 respondents was conducted first. Based on the pretest results, the wording of the survey items was revised. Subsequently, a new round of data collection was conducted, yielding 315 valid survey responses, which were included in the final analysis.

However, the possibility that differences between literal and paraphrased translations of the survey items may have influenced participants’ responses cannot be completely ruled out. Future research could therefore examine alternative item formulations and translation approaches to further reduce potential wording or framing effects.

Finally, the findings are based on the Chinese public health care reporting context and may not be fully generalizable to other countries or health care systems. Future research should test the proposed model across different cultural and institutional contexts.

### Conclusion

This study examined how cognitive, heuristic, and affective information-processing mechanisms shape users’ intentions to use health care PR systems. Contrary to the hypothesized negative relationship, NERs were positively associated with IU. This finding should not be interpreted as indicating that negative emotions generally promote system adoption. Rather, within the context examined in this study, NER may coexist with users’ continued engagement with health care information.

PIA positively influenced VIP, which, in turn, enhanced HIC and IC. IC was the strongest direct predictor of intention to use, while HT also had a positive influence on IU. Contrary to the hypothesized negative relationship, NERs were positively associated with the IU. This finding suggests that NERs do not necessarily inhibit system adoption and, in this context, may be positively associated with users’ intention to engage with health care information.

Overall, the findings highlight that PR system adoption involves cognitive, heuristic, and affective information-processing mechanisms rather than rational information processing alone. The main implication is that PR systems should be designed to support accessible information presentation, comprehensible and credible health care information, and users’ cognitive and NERs.

## Supplementary material

10.2196/105637 Multimedia Appendix 1 Constructs, measurement items, and sources.

10.2196/105637 Multimedia Appendix 2 Partial least squares structural equation modeling assessment results and mediation analysis.

## Abbreviations

AVE: average variance extracted
CR: composite reliability
ELM: elaboration likelihood model
HIC: health information comprehension
HT: heuristic trust
HTMT: heterotrait-monotrait
IC: information credibility
IU: intention to use
LM-RMSE: linear model root mean square error
NER: negative emotional response
PIA: perceived information accessibility
PLS-RMSE: partial least squares root mean square error
PLS-SEM: partial least squares structural equation modeling
PR: public reporting
RQ: research question
SEM: structural equation modeling
TENK: Tutkimuseettinen neuvottelukunta
VIF: variance inflation factor
VIP: visual information presentation
