# Supplementary material for: Information Processing Mechanisms Associated With Intention to Use Public Reporting Systems: Cross-Sectional Study
Source: JMIR Form Res. 2026 Nov 11;10:e105637. doi: 10.2196/105637 (PMC13754263; doi:10.2196/105637)
Supplement: Supplementary file 1 [file formative-v10-e105637-s001.docx]

**Multimedia appendix 1.** Constructs, Measurement Items, and Sources

| **Construct** | **Measurement Items** | **Sources** |
| --- | --- | --- |
| PIA | PIA 1 - I can easily find information on the public reporting website. | Wang and Strong [1]; DeLone and McLean [2] |
|  | PIA 2 - The website’s page categorization and menu design help me locate information. |  |
|  | PIA 3 - Medical data on the website are presented in an accessible manner. |  |
|  | PIA 4 - I can conveniently obtain the medical information I need. |  |
|  | PIA 5 - The organization of information on the website facilitates browsing and searching. |  |
|  | PIA 6 - Important medical information can be directly accessed from the homepage. |  |
| HIC | HIC 1 - I am able to understand the medical information presented on the website. | Nutbeam [3]; Sørensen et al. [4] |
|  | HIC 2 - The medical information on the website is easy for me to understand. |  |
|  | HIC 3 - I am able to understand ratings, statistics, and comparative information presented on the website. |  |
|  | HIC 4 - The explanations provided on the website help me understand the medical data. |  |
| VIP | VIP 1 - Visual elements such as charts, colors, and ratings help me understand medical information. | Card et al. [5]; Lee et al. [6] |
|  | VIP 2 - Star ratings help me compare healthcare providers more easily. |  |
|  | VIP 3 - Visualized information reduces the effort required to understand medical data. |  |
|  | VIP 4 - The visual presentation of medical data is clear and easy to understand. |  |
|  | VIP 5 - Visual information presentation reduces my confusion. |  |
| HT | HT 1 - I tend to trust healthcare providers with higher ratings. | Petty and Cacioppo [7]; Chaiken [8] |
|  | HT 2 - I judge healthcare providers based on overall impressions. |  |
|  | HT 3 -- Good visual presentation enhances my trust in the system. |  |
|  | HT 4 -- Online reviews influence my perception of healthcare quality. |  |
|  | HT 5 - I often make judgments based on easily accessible information. |  |
| IC | IC 1 - I believe that the medical information provided by public reporting websites is reliable. | Hovland and Weiss [9]; Flanagin & Metzger [10] |
|  | IC2 – The data sources of public reporting websites are credible. |  |
|  | IC3 – The medical information on public reporting websites is objective. |  |
|  | IC4 – I believe that the information on public reporting websites is based on real data. |  |
|  | IC5 – I do not perceive obvious bias or distortion in the information provided by public reporting websites. |  |
| NER | NER1 – I feel frustrated when information is difficult to access. | Davis [11]; Chimonas [12]; Shaller [13]; Donovan-Kicken [14] |
|  | NER2 – I feel disappointed when the information provided by the public reporting website does not meet my expectations. |  |
|  | NER3 – I feel frustrated when I cannot quickly find the information I need. |  |
|  | NER4 – Medical information (especially health risk-related information) causes me anxiety. |  |
|  | NER 5 - Emotional responses influence my continued use of public reporting websites. |  |
|  | NER 6 - Excessive medical information makes me feel stressed or overwhelmed. |  |
|  | NER7 – I feel uncertain when medical information is difficult to understand. |  |
| IU | IU 1 - I am willing to use public reporting websites when selecting healthcare providers. | Davis [11]; Ajzen [15] |
|  | IU2 - I will use public reporting systems in future healthcare decisions. |  |
|  | IU3 - I am willing to recommend public reporting websites to others. |  |
|  | IU4 - I believe public reporting information is helpful for healthcare decision-making. |  |
|  | IU5 - I am willing to rely on public reporting systems when comparing healthcare providers. |  |

References:

1.	Wang RY, Strong DM. Beyond accuracy: What data quality means to data consumers. Journal of management information systems 1996;12(4):5-33.

2.	DeLone WH, McLean ER. The DeLone and McLean model of information systems success: a ten-year update. Journal of management information systems 2003;19(4):9-30.

3.	Nutbeam D. Health literacy as a public health goal: a challenge for contemporary health education and communication strategies into the 21st century. Health Promotion International 2000;15(3):259-267. doi: 10.1093/heapro/15.3.259

4.	Sørensen K, Van den Broucke S, Fullam J, Doyle G, Pelikan J, Slonska Z, et al. Health literacy and public health: a systematic review and integration of definitions and models. BMC Public Health 2012;12(1):80.

5.	Card SK, Mackinlay J, Shneiderman B. Readings in information visualization: using vision to think: Morgan Kaufmann; 1999. ISBN: 1558605339.

6.	Lee YW, Strong DM, Kahn BK, Wang RY. AIMQ: a methodology for information quality assessment. Information & management 2002;40(2):133-146.

7.	Petty RE, Cacioppo JT. The elaboration likelihood model of persuasion. In: editors. Adv. Exp. Soc. Psychol. Elsevier; 1986;123-205.

8.	Chaiken S. Heuristic versus systematic information processing and the use of source versus message cues in persuasion. Journal of personality and social psychology 1980;39(5):752.

9.	Hovland CI, Weiss W. The influence of source credibility on communication effectiveness. Public Opinion Quarterly 1951;15(4):635-650.

10.	Flanagin AJ, Metzger MJ. Perceptions of Internet information credibility. Journal. Mass Commun. Q. 2000;77(3):515-540.

11.	Davis FD. Perceived usefulness, perceived ease of use, and user acceptance of information technology. MIS quarterly 1989:319-340.

12.	Chimonas S, Fortier E, Li DG, Lipitz-Snyderman A. Facts and fears in public reporting: patients’ information needs and priorities when selecting a hospital for cancer care. Sage Medical Decision Making 2019;39(6):632-641. doi: doi:10.1177/0272989X19855050

13.	Shaller D, Kanouse DE, Schlesinger M. Context-based strategies for engaging consumers with public reports about health care providers. Medical Care Research and Review 2013;71(5):17S-37S. doi: <https://doi.org/10.1177/1077558713493118>

14.	Donovan-Kicken E, Mackert M, Guinn TD, Tollison AC, Breckinridge B. Sources of patient uncertainty when reviewing medical disclosure and consent documentation. Patient Education and Counseling 2013;90(2):254-260.

15.	Ajzen I. The theory of planned behavior. Organizational behavior and human decision processes 1991;50(2):179-211.
