# Supplementary material for: Information Processing Mechanisms Associated With Intention to Use Public Reporting Systems: Cross-Sectional Study
Source: JMIR Form Res. 2026 Nov 11;10:e105637. doi: 10.2196/105637 (PMC13754263; doi:10.2196/105637)
Supplement: Supplementary file 2 [file formative-v10-e105637-s002.docx]

**Multimedia Appendix 2. PLS-SEM Assessment Results and Mediation Analysis**

**Table 1.** Full Collinearity Variance Inflation Factors.

|  | PIA | HIC | IC | NER | HT | IU | VIP |
| --- | --- | --- | --- | --- | --- | --- | --- |
| PIA |  | 1.469 | 1.456 | 1.516 | 1.485 | 1.518 | 1.500 |
| HIC | 1.707 |  | 1.750 | 1.728 | 1.734 | 1.700 | 1.741 |
| IC | 1.829 | 1.891 |  | 1.883 | 1.906 | 1.736 | 1.841 |
| NER | 1.625 | 1.593 | 1.607 |  | 1.597 | 1.585 | 1.594 |
| HT | 1.831 | 1.840 | 1.871 | 1.837 |  | 1.792 | 1.752 |
| IU | 2.074 | 1.999 | 1.888 | 2.020 | 1.985 |  | 2.041 |
| VIP | 1.908 | 1.905 | 1.864 | 1.891 | 1.807 | 1.900 |  |

**Table 2.** Heterotrait-monotrait ratio (HTMT).

|  | PIA | HIC | IC | NER | HT | IU | VIP |
| --- | --- | --- | --- | --- | --- | --- | --- |
| PIA |  | 0.495 | 0.506 | 0.395 | 0.484 | 0.436 | 0.480 |
| HIC | 0.495 |  | 0.518 | 0.502 | 0.539 | 0.573 | 0.537 |
| IC | 0.506 | 0.518 |  | 0.497 | 0.500 | 0.628 | 0.573 |
| NER | 0.395 | 0.502 | 0.497 |  | 0.514 | 0.542 | 0.523 |
| HT | 0.484 | 0.539 | 0.500 | 0.514 |  | 0.590 | 0.609 |
| IU | 0.436 | 0.573 | 0.628 | 0.542 | 0.590 |  | 0.578 |
| VIP | 0.480 | 0.537 | 0.573 | 0.523 | 0.609 | 0.578 |  |

**Table 3.** Predictive Relevance of the Structural Model.

| Construct | R² | Q²_predict | PLS RMSE | LM RMSE |
| --- | --- | --- | --- | --- |
| VIP | 0.207 | 0.151 | 1.577 | 1.424 |
| HIC | 0.258 | 0.200 | 1.548 | 1.483 |
| IC | 0.244 | 0.192 | 1.528 | 1.397 |
| IU | 0.486 | 0.395 | 1.312 | 1.337 |

**Table 4.** Bootstrapped Confidence Intervals for Mediation Effects.

| Path | O | Mean | 2.5% | 97.5% | 95% CI | Result | Contain 0 |
| --- | --- | --- | --- | --- | --- | --- | --- |
| PIA → VIP → HIC | 0.231 | 0.234 | 0.158 | 0.311 | [0.158, 0.311] | Significant indirect effect | No |
| PIA → VIP → HIC → IC | 0.114 | 0.116 | 0.071 | 0.169 | [0.071, 0.169] | Significant indirect effect | No |
| PIA → VIP → HIC → IC → IU | 0.042 | 0.043 | 0.023 | 0.069 | [0.023, 0.069] | Significant indirect effect | No |
| VIP → HIC → IC | 0.251 | 0.253 | 0.177 | 0.329 | [0.177, 0.329] | Significant indirect effect | No |
| VIP → HIC → IC → IU | 0.092 | 0.093 | 0.057 | 0.137 | [0.057, 0.137] | Significant indirect effect | No |
| HIC → IC → IU | 0.181 | 0.182 | 0.123 | 0.252 | [0.123, 0.252] | Significant indirect effect | No |
